# Phosphorylation regulates proteasomal-mediated degradation and solubility of TAR DNA binding protein-43 C-terminal fragments

**DOI:** 10.1186/1750-1326-5-33

**Published:** 2010-08-30

**Authors:** Yong-Jie Zhang, Tania F Gendron, Ya-Fei Xu, Li-Wen Ko, Shu-Hui Yen, Leonard Petrucelli

**Affiliations:** 1 Department of Neuroscience, Mayo Clinic, 4500 San Pablo Road, Jacksonville, Florida 32224, USA

## Abstract

**Background:**

Inclusions of TAR DNA binding protein-43 (TDP-43) are the defining histopathological feature of several neurodegenerative diseases collectively referred to as TDP-43 proteinopathies. These diseases are characterized by the presence of cellular aggregates composed of abnormally phosphorylated, N-terminally truncated and ubiquitinated TDP-43 in the spinal cord and/or brain. Recent studies indicate that C-terminal fragments of TDP-43 are aggregation-prone and induce cytotoxicity. However, little is known regarding the pathways responsible for the degradation of these fragments and how their phosphorylation contributes to the pathogenesis of disease.

**Results:**

Herein, we established a human neuroblastoma cell line (M17D3) that conditionally expresses an enhanced green fluorescent protein (GFP)-tagged caspase-cleaved C-terminal TDP-43 fragment (GFP-TDP_220-414_). We report that expression of this fragment within cells leads to a time-dependent formation of inclusions that are immunoreactive for both ubiquitin and phosphorylated TDP-43, thus recapitulating pathological hallmarks of TDP-43 proteinopathies. Phosphorylation of GFP-TDP_220-414 _renders it resistant to degradation and enhances its accumulation into insoluble aggregates. Nonetheless, GFP-TDP_220-414 _inclusions are reversible and can be cleared through the ubiquitin proteasome system. Moreover, both Hsp70 and Hsp90 bind to GFP-TDP_220-414 _and regulate its degradation.

**Conclusions:**

Our data indicates that inclusions formed from TDP-43 C-terminal fragments are reversible. Given that TDP-43 inclusions have been shown to confer toxicity, our findings have important therapeutic implications and suggest that modulating the phosphorylation state of TDP-43 C-terminal fragments may be a promising therapeutic strategy to clear TDP-43 inclusions.

## Background

Inclusions of TAR DNA binding protein-43 (TDP-43) are the defining histopathological feature of frontotemporal lobar degeneration with ubiquitin-positive inclusions (FTLD-U) and amyotrophic lateral sclerosis (ALS) [1,2]. Under physiological conditions, TDP-43 predominantly localizes to the nucleus. However, a substantial loss of nuclear TDP-43 is observed in neurons bearing aberrant cytoplasmic TDP-43 inclusions. TDP-43 exhibits a disease-specific biochemical signature; pathologically altered TDP-43 is ubiquitinated, phosphorylated and cleaved to generate C-terminal fragments of 24-26 kDa [1,2].

Recent findings have shown that TDP-43 C-terminal fragments form cytoplasmic aggregates and cause cytotoxicity [3-6]; thus, TDP-43 truncation may play an important role in the pathogenesis of ALS, FTLD-U and other TDP-43 proteinopathies. TDP-43 is a substrate of caspases, as shown by our and others' work, suggesting that caspase-cleaved TDP-43 may account for some of the C-terminal fragments observed in disease [7-9]. Furthermore, three other C-terminal fragments (amino acid residues 208-414, 219-414 and 247-414) have been identified in FTLD-U brain tissue [4,5]. Although the cleavage sites of these reported C-terminal TDP-43 fragments are not identical, they may share similar pathological properties. Ectopic expression of TDP-43 C-terminal fragments in cell culture systems induces cytotoxicity [3] and recapitulates pathological features of disease, including TDP-43 ubiquitination, phosphorylation and cytoplasmic aggregation [3-5]. Of particular interest, the ubiquitination of C-terminal TDP-43 fragments suggests that they are degraded through the ubiquitin-proteasome system (UPS). Despite recent studies that support the notion that full-length and cleaved TDP-43 are degraded via the UPS as well as by autophagy [10-12], our understanding of TDP-43 clearance remains limited.

The hyperphosphorylation of aggregated proteins is a common feature of many neurodegenerative diseases. For instance, the microtubule-associated protein tau is abnormally phosphorylated in Alzheimer's disease as is α-synuclein in Parkinson's disease. It is believed that an imbalance of kinase and phosphatase activity contributes to the abnormal phosphorylation state of tau, which impairs the normal functioning of tau while inhibiting its degradation and facilitating its assembly into paired helical filaments [13]. With regards to TDP-43, little is currently known regarding how phosphorylation affects TDP-43 degradation and aggregation. Recently, it has been shown that the *in vitro *phosphorylation of recombinant full-length TDP-43 by casein kinases enhances TDP-43 oligomerization and fibrillization [14]. However, we and others have demonstrated that phosphorylation of TDP-43 C-terminal fragments at disease-specific sites is not necessary for inclusion formation in cells [3,9]. Even though phosphorylation does not appear to be a requirement for TDP-43 aggregation, it is not yet known if it would accelerate aggregate formation in cells as it does *in vitro*.

To bridge this gap in our understanding, we generated a human neuroblastoma cell line (M17D3) that conditionally expresses an enhanced green fluorescent protein (GFP)-tagged caspase-cleaved C-terminal TDP-43 fragment (GFP-TDP_220-414_), and we examined how the phosphorylation state of GFP-TDP_220-414 _impacts its solubility, aggregation and degradation. We found that the gradual expression of GFP-TDP_220-414 _within cells caused the formation of cytoplasmic inclusions that were immunoreactive for both ubiquitin and phosphorylated TDP-43. Of great significance, we found that these inclusions could be cleared through the UPS, although phosphorylation of TDP-43 C-terminal fragments delayed their degradation. Knocking-down the expression of heat shock proteins (Hsp), Hsp70 or Hsp90, impaired the clearance of GFP-TDP_220-414 _and led to the preferential accumulation of phosphorylated species, which suggests that the Hsp90/Hsp70-based chaperone machinery regulates the degradation of phosphorylated C-terminal TDP-43 fragments. Our findings provide novel insight into understanding how phosphorylation affects the degradation and aggregation of TDP-43 C-terminal fragments. Furthermore, given that TDP-43 inclusions have been shown to confer toxicity [3,6,15], our evidence that such inclusions can be cleared from cells has important therapeutic implications.

## Results

### TDP-43 C-terminal fragments share similar pathological properties

**Figure 1:**
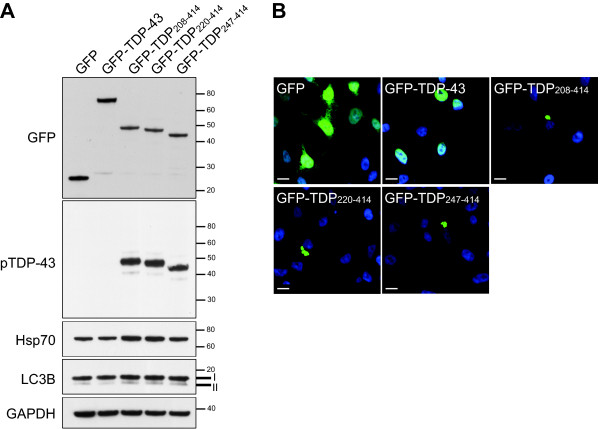
**TDP-43 C-terminal fragments share similar pathological properties, such as phosphorylation and aggregation**. M17 cells were transiently transfected to express GFP, GFP-TDP-43 or various GFP-TDP-43 C-terminal fragments for 2 days. (A) Western blotting of cell lysates using a phosphorylation-dependent (pS409/pS410) TDP-43 antibody (termed pTDP-43) or antibodies towards GFP, Hsp70, LC3B and GAPDH; the latter antibody was used to verify protein loading. (B) GFP fluorescence in cells expressing GFP, GFP-TDP-43 or GFP-tagged C-terminal fragments of TDP-43 was viewed by fluorescent confocal microscopy. Scale bar, 10 μM. Figures shown are representative of the results obtained from 2 independent experiments.

In TDP-43 proteinopathies, TDP-43 is cleaved to generate C-terminal fragments [1]. Given that TDP-43 truncation and phosphorylation are only observed in affected brain and spinal cord regions, these modifications are believed to contribute to the pathogenesis of disease. To test whether various TDP-43 fragments share similar pathological properties in cells, three TDP-43 C-terminal fragments tagged at the amino terminal with enhanced GFP (GFP-TDP_208-414_, GFP-TDP_220-414 _and GFP-TDP_247-414_) were transiently expressed in human neuroblastoma M17 cells and compared to the expression of full-length TDP-43. The transient expression of GFP alone, full-length TDP-43 and all three fragments resulted in a similar level of protein expression (Figure 1A). Full-length GFP-TDP-43 was expressed diffusely within the nucleus of cells whereas the C-terminal TDP-43 fragments formed compact cytoplasmic inclusions (Figure 1B), which is consistent with previous reports [3-5]. We observed similar inclusions following the transient expression of untagged TDP_220-414 _(not shown), indicating that inclusion formation was not merely due to the GFP tag. Note that only TDP-43 fragments were phosphorylated at pathologically specific sites (serine 409 and serine 410) [14]. Of interest, the expression of GFP-TDP_208-414_, GFP-TDP_220-414 _and GFP-TDP_247-414_, but not that of GFP-TDP-43, appeared to moderately induce the molecular chaperone Hsp70 (Figure 1A), suggesting that inclusion formation may induce a stress response in cells. In contrast, no obvious change in the ratio between uncleaved (LC3B-I) and cleaved (LC3B-II) LC3B, a common indicator of autophagy [16], was observed among samples (Figure 1A). Based on these findings, we generated a stable cell line that conditionally expresses the C-terminal fragment corresponding to caspase-cleaved TDP-43 (GFP-TDP_220-414_) in order to study how phosphorylation influences degradation and inclusion formation of TDP-43 C-terminal fragments.

### Inducible expression of TDP-43 C-terminal fragment recapitulates pathological hallmarks of TDP-43 proteinopathies

**Figure 2:**
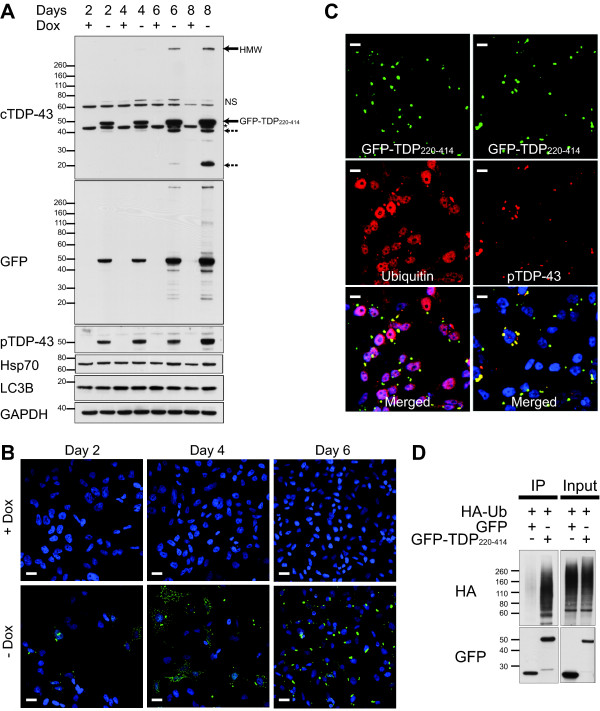
**Time-dependent expression and aggregation of GFP-TDP_220-414 _in M17D3 cells**. M17D3 cells were cultured in media lacking or containing 1 μg/ml doxycycline to either induce or suppress the expression of GFP-TDP_220-414_, respectively. (A) The expression of total and phosphorylated-GFP-TDP_220-414 _were assessed using antibodies towards the C-terminus of TDP-43 (cTDP-43) or anti-pTDP-43, respectively. Endogenous TDP-43 is indicated by an asterisk (*) while dashed arrows indicate TDP-43 fragments, which likely resulted from the truncation of GFP-TDP_220-414_. HMW=high-molecular weight, NS = non-specific band, Dox=doxycycline. (B) GFP fluorescence in cells expressing GFP-TDP_220-414 _was viewed by fluorescent confocal microscopy at 2, 4 and 6 days post-induction and in non-induced controls. Scale bar, 20 μM. (C) GFP-TDP_220-414_-expressing cells were immunostained with antibodies towards S409/S410-phosphorylated TDP-43 or ubiquitin. Scale bar, 10 μM. Figures shown are representative of the results obtained from 4 independent experiments. (D) M17 founder cells were cotransfected for 48 h with HA-ubiquitin and GFP or GFP-TDP_220-414_. Cell lysates were incubated with anti-GFP. Protein G agarose was added to capture the protein-antibody complex and then the captured protein was eluted from the beads and resolved by SDS-PAGE for Western blot analysis. Blots were probed with antibodies towards the HA-tag or GFP. Figures shown are representative of the results obtained from 3 independent experiments.

By using a tetracycline-off inducible gene expression system, we produced a stable human neuroblastoma cell line (termed M17D3) that inducibly expresses GFP-TDP_220-414 _in the absence of doxycycline, a tetracycline derivative. No GFP-TDP_220-414 _was expressed in cells when doxycycline was present (Figure 2A and 2B), confirming that GFP-TDP_220-414 _expression was tightly regulated. To test whether gradual expression of GFP-TDP_220-414 _mimics the aggregation process in disease, M17D3 cells were grown in doxycycline-free media for 2, 4, 6 or 8 days; this led to a time-dependent expression of GFP-TDP_220-414 _(Figure 2A). GFP-TDP_220-414 _was phosphorylated at S409/S410 as early as 2 days post-induction, with levels increasing noticeably by day 8 (Figure 2A). By 6 days of induction, high-molecular weight (HMW) TDP-43-immunoreactive products were observed. These HMW species may be a product of GFP-TDP_220-414 _oligomerization or the result of other types of modifications, such as cross-linking (Figure 2A). The appearance of the HMW products and the formation of large cytoplasmic inclusions (Figure 2B) coincided with an increase in the expression of Hsp70 (Figure 2A). Cleavage of LC3B, however, was not evident at any time-point post-induction (Figure 2A).

Fluorescent confocal microscopy revealed that numerous small cytoplasmic inclusions were observed within cells as early as 2 days following the induction of GFP-TDP_220-414 _(Figure 2B). The lack of diffuse GFP fluorescence at this time-point highlights the high propensity for GFP-TDP_220-414 _to aggregate upon expression. At later time-points, larger, but fewer, inclusions were present per cell, suggesting that the larger inclusions were formed by the assembly of small inclusions (Figure 2B). In addition, the larger inclusions at day 6 were intensely stained by antibodies for ubiquitin and pTDP-43 (Ser409/410), suggesting that GFP-TDP_220-414 _within inclusions is phosphorylated and ubiquitinated (Figure 2C). To definitively determine if GFP-TDP_220-414 _is ubiquitinated, cells were transiently co-transfected to express HA-ubiquitin and GFP-TDP_220-414 _or GFP. Two days post-transfection, a GFP-antibody was used to immuoprecipitate GFP-TDP_220-414 _or GFP from cell lysates. Although both GFP and GFP-TDP_220-414 _were pulled down efficiently under these experimental conditions, only GFP-TDP_220-414 _was ubiquitinated (Figure 2D). These findings indicate that inducible expression of GFP-TDP_220-414 _recapitulates pathological hallmarks of TDP-43 proteinopathies.

### TDP-43 C-terminal fragment is preferentially degraded through the ubiquitin-proteasome system

**Figure 3:**
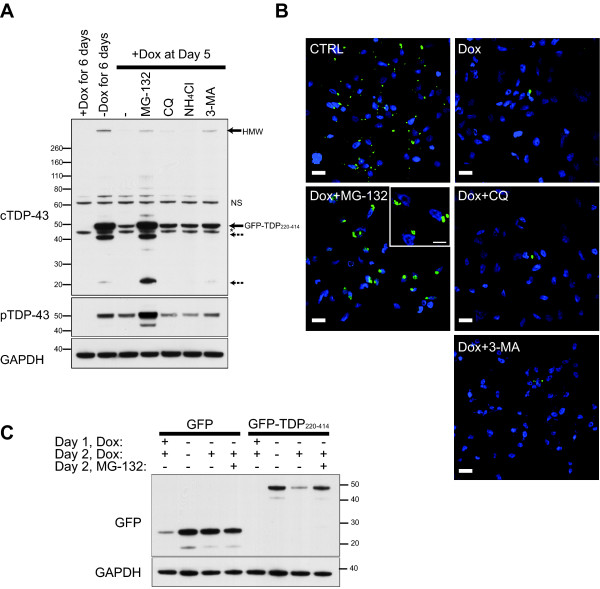
**The TDP-43 C-terminal fragment, GFP-TDP_220-414_, is preferentially degraded through the ubiquitin-proteasome pathway**. To monitor GFP-TDP_220-414 _degradation, M17D3 were grown in doxycycline-free media for 5 days to induce GFP-TDP_220-414 _expression. Its expression was then inhibited by doxycycline and cells were treated with the proteasome inhibitor, MG-132, the lysosome inhibitors, chloroquine (CQ) or NH_4_Cl, or the autophagy inhibitor, 3-MA. Control cultures were maintained in doxycycline-free media to sustain GFP-TDP_220-414 _expression. On day 6, cells were harvested for Western blot analysis and confocal microscopy. (A) Blots were probed using an anti-cTDP-43, anti-pTDP-43 and anti-GAPDH antibodies. The arrow indicates GFP-TDP_220-414_, the asterisk (*) endogenous TDP-43, and the dashed arrows cleavage products, likely resulting from GFP-TDP_220-414 _truncation. Dox=doxycycline, HMW = high molecular weight, NS=non-specific. (B) GFP fluorescence in cells after 6 days of GFP-TDP_220-414 _induction in doxycycline-free media (CTRL) was compared to that in cells treated with doxycycline during the last 24 h of the 6-day experiment (Dox) or to cells co-treated with doxycycline and MG-132 (Dox+MG-132), chloroquine (Dox+CQ), or 3-MA (Dox+3-MA). Scale bars, 20 μM (10 μM in inset). (C) To determine if the GFP-tag is responsible for targeting GFP-TDP_220-414 _to the proteasome for degradation, M17D cells were transfected with pTRE-GFP or pTRE-GFP-TDP_220-414 _for 2 days. During these 2 days, cultures were treated with or without doxycycline and MG-132, as indicated. Cells were then harvested for Western blot analysis using anti-GFP and anti-GAPDH. GFP-TDP_220-414, _but not GFP, was degraded by the proteasome. Figures shown are representative of the results obtained from 3 independent experiments.

To monitor GFP-TDP_220-414 _clearance and to assess if inclusions, once formed, can be degraded, cells were induced to express GFP-TDP_220-414 _for 5 days and then exposed to doxycycline to block transgene expression for 1 day thereafter. As a control, sister-cultures were grown in doxycycline-free media to maintain GFP-TDP_220-414 _expression for the duration of the 6-day experiment. As expected, GFP-TDP_220-414 _was highly expressed (Figure 3A), and many inclusions were present in these control cultures (Figure 3B). Conversely, a marked decrease in protein level of GFP-TDP_220-414 _(Figure 3A) and in the number of inclusion-bearing cells (Figure 3B) were observed in cultures that were treated with doxycycline on day 5. The degradation of GFP-TDP_220-414 _was completely suppressed by the proteasome inhibitor, MG-132 (Figure 3A and 3B). Interestingly, inclusions in MG-132-treated cells were larger than those in control cells, suggesting that blocking the degradation of GFP-TDP_220-414_, and consequently promoting the accumulation of phosphorylated GFP-TDP_220-414_, increases the rate of inclusion assembly. In contrast to MG-132, neither the lysosomal inhibitors, chloroquine and NH_4_Cl, nor the autophagy inhibitor, 3-MA, appreciably blocked the degradation of GFP-TDP_220-414 _(Figure 3A and 3B) indicating that this TDP-43 C-terminal fragment is preferentially degraded via the UPS.

To determine whether the targeting of GFP-TDP_220-414 _to the proteasome for degradation was dependent upon its fusion to GFP, M17D Tet-Off founder cells were made to transiently express GFP-TDP_220-414 _or GFP. Compared to cells expressing GFP-TDP_220-414 _for 2 days, the level of GFP-TDP_220-414 _was noticeably decreased in cells treated with doxycycline to suppress GFP-TDP_220-414 _expression during the second day of the 2-day experiment (Figure 3C). However, if the proteasome was inhibited by MG-132, GFP-TDP_220-414 _degradation was suppressed (Figure 3C). In contrast, GFP levels remained unchanged even when GFP expression was blocked during the second of the 2 day experiment (Figure 3C). These results indicate that the proteasomal degradation of GFP-TDP_220-414 _is not due to the targeting of GFP to the proteasome.

### Knockdown of heat shock proteins leads to the accumulation of TDP-43 C-terminal fragment

**Figure 4:**
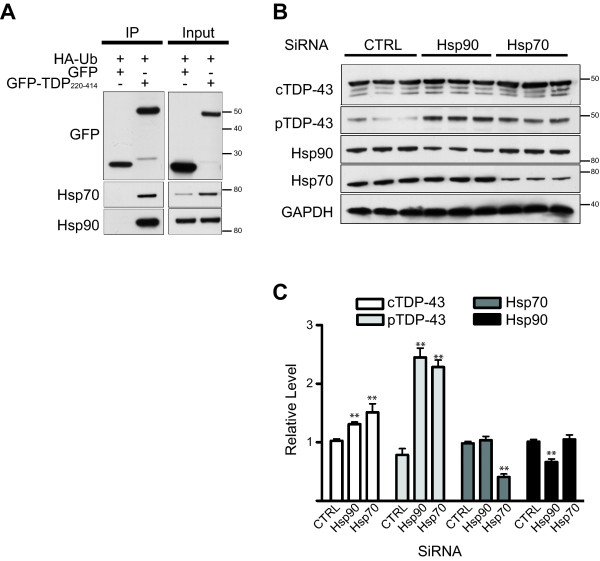
**Knockdown of heat shock proteins leads to the accumulation of TDP-43 C-terminal fragments**. (A) Co-immunoprecipitation studies showed that GFP-TDP_220-414_, but not GFP, binds to Hsp70 and Hsp90. M17 founder cells expressing HA-ubiquitin and GFP or GFP-TDP_220-414 _were harvested and lysates were incubated with anti-GFP. Protein G agarose was added to capture the protein-antibody complex and the captured protein was eluted from the beads and resolved by SDS-PAGE for Western blot analysis. Blots were probed with antibodies towards GFP, Hsp70 or Hsp90 (note that blots were also probed with an antibody toward the HA-tag, which is shown in Figure 2D). Figures shown are representative of the results obtained from 3 independent experiments. (B-C) To explore the role of Hsp70 and Hsp90 in the degradation of GFP-TDP_220-414_, M17D3 cells were grown in doxycycline-free medium for 4 days then treated with siRNA targeted to Hsp90 or Hsp70 or with a validated negative siRNA control. After 48 h, cells were harvested for Western blot analysis using the indicated antibodies. Levels of total TDP-43, phosphorylated TDP-43, Hsp70 and Hsp90 were determined by densitometric analysis (C). Data was collected from 3 separate experiments and shown as the mean ± SEM. ** represents *P *< 0.001, as assessed by 1-way ANOVA, followed by Tukey's posthoc analysis.

Hsp70 and Hsp90 play critical roles in protein quality control. In Alzheimer's disease and Huntington's disease, these chaperones bind to tau or mutant huntington protein, respectively, and target these aggregation-prone proteins to the proteasome for degradation [17,18]. Given these findings and the fact that Hsp70 protein levels were increased by the expression of TDP-43 C-terminal fragments (Figs. 1A and 2A), we tested whether Hsp90 and Hsp70 bind GFP-TDP_220-414 _and mediate its degradation. Immuoprecipitation studies indicated that GFP-TDP_220-414_, but not GFP, bind Hsp70 and Hsp90 (Figure 4A). In order to investigate if the proteasomal degradation of GFP-TDP_220-414 _is mediated by its interaction with Hsp90 or Hsp70, cells were incubated in doxycycline-free media for 4 days followed by a 2 day treatment with siRNAs targeted to Hsp90 or Hsp70. This led to a decrease in Hsp90 and Hsp70 protein expression by 34% and 60%, respectively (Figure 4B and 4C). Decreasing Hsp70 or Hsp90 expression resulted in increased levels of GFP-TDP_220-414_, as assessed using both phosphorylation-independent and -dependent TDP-43 antibodies (Figure 4B). Compared to GFP-TDP_220-414 _in cells treated with non-silencing siRNA, total and phosphorylated GFP-TDP_220-414 _levels were elevated by approximately 1.3-fold and 2.8-fold, respectively, in cells treated with Hsp90 siRNA (Figure 4C). In a similar fashion, Hsp70 siRNA treatment led to 1.5-fold and 2.5-fold increase in total and phosphorylated GFP-TDP_220-414_, respectively (Figure 4C). More important, compared to the 30-50% increase in total GFP-TDP_220-414 _resulting from Hsp90 or Hsp70 knockdown, phosphorylated-GFP-TDP_220-414 _levels were significantly increased by nearly 150-180% (Figure 4C). These results suggest that Hsp70 and Hsp90 preferentially facilitate the degradation of phosphorylated C-terminal fragments and that impairment of the Hsp70/Hsp90/GFP-TDP_220-414 _complex leads to the accumulation of phosphorylated-GFP-TDP_220-414_.

### Phosphorylated TDP-43 C-terminal fragment is resistant to proteasomal degradation

**Figure 5:**
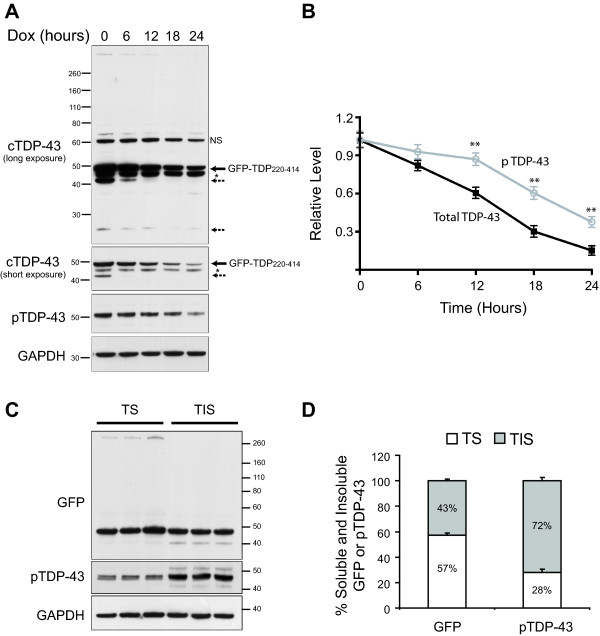
**Phosphorylated TDP-43 C-terminal fragment is resistant to proteasomal degradation**. To calculate the half-life of GFP-TDP_220-414_, cells were maintained in doxycycline-free media for 5 days. Doxycycline (1 μg/ml) was then added to the media to arrest GFP-TDP_220-414 _expression. Cells were harvested immediately (0 hours) as well as 6, 12, 18 and 24 hours after the addition of doxycycline. (A) Western blot analysis of cell lysates using antibodies towards the C-terminus of TDP-43 (cTDP-43) or phosphorylated TDP-43 (pTDP-43). The arrow indicates GFP-TDP_220-414_, the asterisk (*) endogenous TDP-43, and the dashed arrows cleavage products, likely generated from the truncation of GFP-TDP_220-414_._. _Dox=doxycycline, NS=non-specific. (B) Densitometric quantification of GFP-TDP_220-414 _in cells was calculated by dividing the density of bands for total or phosphorylated GFP-TDP_220-414 _by that of the corresponding GAPDH band, then normalizing each time-point to GFP-TDP_220-414_/GAPDH levels at 0 hours. The half life (T1/2) of total GFP-TDP_220-414 _was 14.2 hours; T1/2 for phospho-GFP-TDP_220-414 _was 22.1 hours. ** represents *P*<0.001, as assessed by 1-way ANOVA, followed by Tukey's posthoc analysis (n = 3). (C) To examine the solubility of total and phosphorylated-GFP-TDP_220-414_, M17D3 cells were incubated in doxycycline-free media for 6 days. Cell lysates were separated into Triton X-100-soluble (TS) and -insoluble (TIS) fractions and analyzed by Western blotting using anti-GFP and anti-pTDP-43 antibodies. (D) Densitometric quantification of total and phosphorylated GFP-TDP_220-414 _in the Triton X-100-soluble and -insoluble fractions. Data was collected from 3 separate experiments and is shown as the mean+SEM.

To further assess how phosphorylation influences the degradation of truncated TDP-43, GFP-TDP_220-414 _was inducibly expressed for 5 days, at which point 1 μg/ml doxycycline was added to the medium to halt transgene expression. Cells were harvested at 0, 6, 12, 18 or 24 hours after the addition of doxycycline for Western blot analysis of total GFP-TDP_220-414 _and phosphorylated-GFP-TDP_220-414 _(Figure 5A). The amount of total or phosphorylated GFP-TDP_220-414 _remaining in the lysates at each time-point was normalized to the amount present at the time doxycycline was added (t = 0). Of interest, phosphorylated-GFP-TDP_220-414 _was cleared from cells more slowly than total GFP-TDP_220-414 _(t1/2 = 22.1 hours vs. 14.2 hours, respectively; Figure 5A and 5B).

To characterize the solubility of phosphorylated-GFP-TDP_220-414_, cell lysates were separated into Triton X-100-soluble and -insoluble fractions and analyzed by Western blotting. While the majority of total GFP-TDP_220-414 _(~57%) was concentrated in the detergent soluble fraction (Figure 5C and 5D), the opposite was observed for phosphorylated-GFP-TDP_220-414. _Seventy-two percent of phosphorylated-GFP-TDP_220-414 _accumulated in the detergent insoluble fraction (Figure 5C and 5D). These findings suggest that phosphorylation renders GFP-TDP_220-414 _resistant to proteasomal degradation and decreases its solubility.

## Discussion

In ALS, FTLD-U, as well as other TDP-43 proteinopathies, the presence of TDP-43-positive inclusions within affected neurons and glia is accompanied by abnormal TDP-43 ubiquitination, phosphorylation and cleavage to generate C-terminal fragments [1]. Since C-terminal fragments of TDP-43 are recovered in the sarkosyl-insoluble fraction of brain and spinal cord homogenates of diseased patients, it has been hypothesized that they serve to seed TDP-43 aggregation and play an important role in disease pathogenesis. Recently, we and others have demonstrated that transient expression of TDP-43 C-terminal fragments within cells causes the formation of cytoplasmic inclusions [3-5]. Such an approach results in very high expression levels of the exogenous protein during a relatively short time period. In the present study, we used a tetracycline-inducible model to determine whether a more gradual expression of caspase-cleaved TDP-43 C-terminal fragments (GFP-TDP_220-414_) also leads to inclusion formation. By slowing the time-course over which inclusions are formed, we were able to investigate the aggregation process in more detail. It is important to note that numerous small cytoplasmic inclusions were observed within cells as early as 2 days following the induction of GFP-TDP_220-414 _(Figure 2). Minor diffuse GFP fluorescence was observed at this time-point underscoring the high propensity of GFP-TDP_220-414 _to aggregate upon expression. At later time-points, larger, but fewer, inclusions were present per cell, indicating that large inclusions are formed by the assembly of small inclusions (Figure 2). The presence of large cytoplasmic inclusions at day 6, as assessed by immunofluorescence studies, coincide with the induction of Hsp70, as assessed by Western Blot (Figure 2), suggesting that inclusion formation induces a heat shock response. Since we found that Hsp70, as well as Hsp90, play a role in regulating GFP-TDP_220-414 _degradation (Figure 4), the induction of Hsp70 may be an attempt made by cells to promote the clearance of GFP-TDP_220-414 _once levels reach a harmful threshold. Indeed, it is becoming increasingly apparent that C-terminal TDP-43 fragments are toxic. We have previously shown that the aggregation of these fragments is associated with increased cytotoxicity, which likely results from a toxic gain of function since GFP-TDP_220-414 _inclusion formation neither alters the nuclear distribution of endogenous full-length TDP-43, nor does it reduce its function, as assessed using an exon skipping assay [3]. In addition, studies conducted in yeast have shown that TDP-43 species that form aggregates are toxic and that the C-terminal region of TDP-43 is required for both aggregation and toxicity [6].

The ubiquitination of TDP-43 inclusions in disease [1,2] suggests that full-length and/or truncated TDP-43 are substrates of the UPS. Consistent with our findings, recent studies have shown that full-length TDP-43 and TDP-43 fragments are ubiquitinated [11,12] and can be cleared by the UPS [11,12] and autophagy [10-12] in cultured cells. However, the earlier studies did not examine the molecular determinants of TDP-43 degradation, such as TDP-43 phosphorylation, as done in the present study. To better understand the degradation pathways of C-terminal TDP-43 fragments, we took advantage of our inducible cell model and the fact that transcription of GFP-TDP_220-414 _could be inhibited by the addition of doxycycline. The latter point is important since studies have shown that proteasomal inhibitors cause transcriptional up-regulation of the cytomegalovirus (CMV) promoter [19,20], which was used to drive expression of TDP-43 products in the present study and the study conducted by Wang et al. (2010), who previously reported that TDP-43 accumulates following proteasomal inhibition [11]. Because doxycycline blocks transcription of GFP-TDP_220-414 _in our inducible cell model, it is ideally suited to assess steady-state levels of GFP-TDP_220-414 _and how GFP-TDP_220-414 _protein levels are affected upon treatment with proteasome inhibitors. Our findings indicate that GFP-TDP_220-414 _was ubiquitinated and markedly accumulated when cells were treated with the proteasome inhibitor, MG-132. In contrast, only modest increases in GFP-TDP_220-414 _were observed following inhibition of the autophagic-lysosomal pathway. The accumulation of GFP-TDP_220-414 _upon proteasome inhibition was specific, since GFP, unlike GFP-TDP_220-414_, did not accumulate during MG-132 treatment. These findings suggest that C-terminal TDP-43 fragments are degraded predominantly through the UPS (Figure 2 and 4).

Most important, our data indicates that, at the time-points examined, inclusion formation is reversible if the overexpression of GFP-TDP_220-414 _is halted. The clearance of inclusions is due to proteasome-mediated degradation of GFP-TDP_220-414 _since proteasomal inhibition, but not lysosomal/autophagy inhibition, prevents inclusion clearance. While both total GFP-TDP_220-414 _and phosphorylated-GFP-TDP_220-414 _were degraded when GFP-TDP_220-414 _was suppressed, it is noteworthy that the half-life of phosphorylated GFP-TDP_220-414 _was longer than that of total GFP-TDP_220-414_. Moreover, the majority of phosphorylated GFP-TDP_220-414 _within cells was insoluble. While we have previously reported that GFP-TDP_220-414 _need not be phosphorylated to aggregate [3], our present findings suggest that phosphorylation of GFP-TDP_220-414 _may promote the formation of insoluble inclusions thus rendering phosphorylated-GFP-TDP_220-414 _resistant to degradation compared to its non-phosphorylated counterpart (Figure 5). Consistent with this, *in vitro *phosphorylation of recombinant full-length TDP-43 by casein kinases was shown to enhance TDP-43 oligomerization and fibrillization [14]. Ample evidence also suggests that covalent modifications of proteins, such as phosphorylation, facilitate protein aggregation in various neurodegenerative diseases. For example, studies report that phosphorylation of α-synuclein, ataxin-1 and tau regulates their aggregation and inclusion formation [21-23].

Of particular interest, we found that knocking-down Hsp90 or Hsp70 led to elevated levels of GFP-TDP_220-414 _and especially phosphorylated-GFP-TDP_220-414 _(Figure 4), suggesting that chaperone-mediated degradation accounts, at least in part, for the clearance of GFP-TDP_220-414_. This is further supported by the fact that GFP-TDP_220-414 _binds both Hsp70 and Hsp90, two molecular chaperones involved in targeting client proteins for proteasome-mediated degradation. Phosphorylated-GFP-TDP_220-414 _accumulated to a greater degree compared to total GFP-TDP_220-414 _following Hsp70 or Hsp90 knockdown (Figure 5). Again, this may suggest that phosphorylated-GFP-TDP_220-414 _is resistant to degradation, such that perturbations in the mechanisms through which it is cleared cause a more appreciable accumulation. However, the significant accumulation of phosphorylated-GFP-TDP_220-414 _compared to total GFP-TDP_220-414 _following Hsp70 or Hsp90 knockdown could also indicate that phosphorylated-GFP-TDP_220-414 _is preferentially targeted by Hsp90 and Hsp70. Indeed, the phosphorylation state of other proteins implicated in neurodegenerative diseases is reported to influence their clearance; for instance, we have shown that chaperone induction results in the selective proteasomal degradation of specific phosphorylated and conformationally altered tau species, while sparing tau phosphorylated at different sites from being degraded [24].

## Conclusions

In summary, the findings of the present study indicate: 1) Gradual expression of GFP-TDP_220-414 _within neuroblastoma cells leads to the formation of cytoplasmic inclusions that are immunoreactive for both ubiquitin and phosphorylated TDP-43; 2) At the time-points examined, the inclusions are reversible and cleared primarily through the UPS; 3) Phosphorylation of TDP-43 C-terminal fragments slows degradation and likely facilitates aggregation; and 4) Hsp70 and Hsp90 regulate GFP-TDP_220-414 _degradation, such that knocking-down Hsp70 or Hsp90 expression causes GFP-TDP_220-414, _and especially phosphorylated-GFP-TDP_220-414, _to accumulate. Our findings provide novel insight regarding the influence of phosphorylation on the degradation and aggregation of TDP-43 C-terminal fragments. Because phosphorylation may hinder TDP-43 degradation and accelerate inclusion formation in disease, modulating the phosphorylation state of TDP-43 truncation products may prove to be a promising therapeutic approach to enhance clearance of TDP-43 inclusions. Indeed, it is most promising that our results indicate that inclusions, once formed, can be reversed. Our novel cell model provides an ideal tool for high-throughput screening of small-molecule libraries for the identification of compounds that diminish TDP-43 inclusions.

## Methods

### Plasmids

The GFP-tagged TDP-43 C-terminal fragments (GFP-TDP_208-414 _and GFP-TDP_247-414_) were generated by PCR to fuse GFP to the 5' end of TDP-43. The primers were: GFP-TDP_208-414_: 5'-CGGGATCCATGCGGGAGTTCTTCTCTCAGTACG-3' and 5'-GCTCTAGACTACATTCCCCAGCCAGAAGAC-3'; GFP-TDP_247-414_: 5'-CGGGATCCGACTTGATCATTAAAGG-3' and 5'-GCTCTAGACATTCCCCAGCCAGAAGAC-3'. The PCR product was subcloned into the pEGFP-C1 vector (Clontech) using restriction sites BamHI and XbaI.

The pTRE-GFP-TDP_208-414_, pTRE-GFP-TDP_2220-414_, pTRE-GFP-TDP_247-414_, pTRE-GFP-TDP-43 or pTRE-GFP plasmids were constructed by cloning cDNA into the pTRE vector using SacII and XbaI restriction sites. Because the SacII restriction site was present within the GFP-TDP_X-X _cDNA, site-directed mutagenesis (Strategene) was used to eliminate the SacII site using the following primers: 5'-GAATTCTGCAGTCGACGGTACAGCAGGCCCGGGATCCATG-3' and 5'-CATGGATCCCGGGCCTGCTGTACCGTCGACTGCAGAATTC-3'. Afterwards, the pTRE-GFP-TDP_X-X _plasmids were generated using primers 5'-TCCCCGCGGCGCCACCATGGTGAGCAAG-3' and 5'-GCTCTAGACTACATTCCCCAGCCAGAAGAC-3'. The pTRE-GFP plasmid was generated by using the primers: 5'-TCCCCGCGGCGCCACCATGGTGAGCAAG-3' and 5'-GCTCTAGACTACTTGTACAGCTCGTCCATG-3'. The pTRE-GFP-TDP_X-X _or pTRE-GFP plasmids conditionally express GFP-tagged full-length TDP-43, TDP-43 C-terminal fragments or GFP upon removal of doxycycline from the media.

### Transient Expression of TDP-43 C-terminal Fragments

To detect if various TDP-43 C-terminal fragments share similar pathological features in cells, M17 founder cells were grown in 24-well or 6-well plates in Opti-Mem supplemented with 10% fetal bovine serum (FBS) and 1% penicillin/streptomycin. When cells reached 90% confluency, they were transfected with 0.3 μg/well (24-well plate) or 1 μg/well (6-well plates) of plasmid using Lipofectamine 2000 (Invitrogen) according to the manufacturer's instructions. After 48 h, the cells were fixed for confocal analysis or harvested for Western blot analysis.

### Generation of Stable Human Neuroblastoma M17 Cell Lines Conditionally Expressing GFP-TDP_220-414_

The human neuroblastoma M17 D tetracycline off (TetOff) founder line has previously been described [25]. M17 D cells were grown in Opti-Mem supplemented with 10% FBS, 1% penicillin/streptomycin, 400 μg/ml G418 and 1 μg/ml doxycycline. For transfection of the pTRE-GFP-TDP_220-414 _plasmid, M17 D cells were seeded in a 10 cm dish. Once 90% confluency was reached, cells were cotransfected with 5 μg pTRE-GFP-TDP_220-414 _and 0.5 μg PVGRX (zeocin-resistant gene) by using Lipofectamine 2000 (Invitrogen) according to the manufacturer's instructions. Forty-eight hours after transfection, the cells were suspended in 5 ml medium, and then suspended cells were diluted 1000 times and seeded in a 10 cm dish. Twenty-four hours later, 100 μg/ml zeocin was added to the medium to screen for clones resistant to zeocin. After incubating cells for 10 days, single clones were picked and seeded into a 24-well plate. When the cell confluency reached ~80%, each clone was split into duplicate wells and cells were grown in Opti-Mem supplemented with 10% FBS, 1% penicillin/streptomycin, 400 μg/ml G418, 1 μg/ml doxycycline and 100 μg/ml zeocin. When cell confluency reached ~80%, doxycycline was removed from one of the duplicate wells to induce transgene expression. Inclusion formation was monitored by fluorescence microscopy. The clone M17D3 was chosen and used throughout the study.

### Cell Treatments

To examine inclusion formation by confocal microscopy and to assess GFP-TDP_220-414 _protein expression by Western blotting, M17D3 cells, seeded at 6.0 × 10^4 ^cells per well in 24-well plates or at 2.4 × 10^5 ^cells per well in 6-well plates, were grown in culture medium in the presence of 1 μg/ml doxycycline to inhibit GFP-TDP_220-414 _expression or in the absence of doxycycline to induce GFP-TDP_220-414 _expression. Cells were fixed or harvested at the indicated time-points for confocal microscopy and Western blot analysis, respectively.

To determine whether inclusions formed during GFP-TDP_220-414 _expression can be degraded when protein expression is arrested, GFP-TDP_220-414 _was inducibly expressed for 5 days. On day 5, 1 μg/ml doxycycline was added to the culture medium to halt GFP-TDP_220-414 _expression. Also added was 10 μM MG-132 (proteasome inhibitor), 50 μM chloroquine (lysosome inhibitor), 10 mM NH_4_Cl (lysosome inhibitor) or 10 mM 3-MA (autophagy inhibitor). On day 6, cells were fixed or harvested for confocal microscopy and Western blot analysis, respectively.

To determine the half-life of GFP-TDP_220-414_, its expression was induced for 5 days, at which point 1 μg/ml doxycycline was added to the medium to halt transgene expression. Cells were harvested at 0, 6, 12, 18 or 24 hours after the addition of doxycycline for Western blot analysis.

To confirm that degradation of GFP-TDP_220-414 _is not simply due to targeted degradation of the GFP tag, M17 founder cells seeded in 6-well plates were transfected with 0.5 μg of pTRE-GFP-TDP_220-414 _or pTRE-GFP plasmid in the presence or absence of 1 μg/ml doxycycline. Twenty-four hours after transfection, 1 μg/ml doxycycline (with or without 10 μM MG-132) was added to the culture medium and cells were harvested for Western blot analysis 24 hours later.

To determine if Hsp90 or Hsp70 knockdown affects GFP-TDP_220-414 _degradation, their expression was reduced by using small interfering RNA (siRNA). Briefly, M17D3 cells, seeded at 2.4 × 10^5 ^cells per well in 6-well plates, were grown in doxycycline-free medium for 4 days. Then, 20 nM/well of siRNA (Hsp90, Hsp70 or a validated negative control siRNA) was transfected into cells using siLentFect transfection reagent (Bio-Rad) according to the manufacturer's instructions. After 48 hours, cells were harvested for Western blot analysis. The siRNA was predesigned by Qiagen. The sense sequence for each were: Hsp90 r(CCGACGAUAUUACUAAUGA)dTdT; Hsp70 r(CCAUUGAGGAGGUAGAUUA)dTdT.

### Immunofluorescence

To determine if inclusions composed of C-terminal fragments are ubiquitinated and phosphorylated, cells grown on coverslips were fixed with 4% paraformaldehyde in phosphate-buffered saline (PBS) at 4 °C for 15 min, and then permeabilized with 0.5% Triton X-100 in PBS for 10 min. After blocking with 5% bovine serum albumin for 1 h at 37 °C, the cells were incubated overnight at 4 °C with rabbit polyclonal ubiquitin antibody (1:100, DakoCytomation) or rabbit polyclonal anti-pTDP-43 (which detects phosphorylated S409/S410; 1:1000, Cosmo Bio Co. Ltd.). After washing, cells were incubated with the Alexa 568-conjugated goat anti-mouse or anti-rabbit IgG secondary antibody (1:1000, Molecular Probes). Hoechst 33258 (1 μg/ml) was used to stain the nuclei and images were obtained on a Zeiss LSM 510 META confocal microscope.

### Co-Immunoprecipitation

For co-immunoprecipitation studies, M17 founder cells were cotransfected with 0.5 μg HA-Ubiquitin and 1 μg GFP or GFP-TDP_220-414_. Cells were harvested 48 h later using Co-IP buffer (50 mM Tris-HCl, pH 7.4, 1 M NaCl, 1% Triton X-100, 5 mM EDTA) containing PMSF as well as protease and phosphatase inhibitors. The lysates were sonicated and centrifuged at 16,000 g for 20 min. The protein concentration of supernatants was determined by BCA assay (Thermo Scientific). Supernatant containing 300 μg of total protein was pre-cleared with 15 μl Protein G agarose (Pierce) for 30 min, then incubated with rabbit polyclonal anti-GFP antibody (0.5 ul, Abcam) overnight at 4 °C with gentle shaking. Next, 15 ul Protein G agarose was added to capture the protein-antibody complex. Following a 6 h incubation at 4 °C, the agarose was pelleted by centrifugation at 1, 000 g for 3 min and washed with Co-IP buffer 6 times. Captured protein was eluted from the beads using sample loading buffer and resolved by SDS/PAGE for Western blot analysis.

### Fractionation Assay

Briefly, cells were lysed in a buffer containing Co-IP buffer plus PMSF and both a protease and phosphatase inhibitor mixture. After sonication, cells were centrifuged at 16,000 g at 4°C for 20 min. Triton X-100-insoluble pellets were dissolved in the Co-IP buffer plus 1% SDS, PMSF, and both a protease and phosphatase inhibitor mixture. The soluble and insoluble fractions were used for Western blot analysis.

### Western Blot Analysis

Cells were lysed in lysis buffer consisting of Co-IP buffer plus 1% SDS, PMSF, and both a protease and phosphatase inhibitor mixture. The protein concentration of cell lysates was measured using a BCA assay (Pierce). Samples were heated in Laemmli's buffer and equal amounts of protein were loaded into 10-well 4-12% Bis-Tris gels (Novex, Invitrogen). After transfer, blots were blocked with 5% nonfat dry milk in TBST (TBS plus 0.1% Triton X-100) for 1 h, and then blots were incubated with rabbit polyclonal cTDP-43 antibody (developed against residues 350-414 of TDP-43; 1: 2000, Abcam), rabbit polyclonal GFP antibody (1:2000, Invitrogen), rabbit polyclonal anti-pTDP-43 (which detects phosphorylated S409/S410; 1:2000, Cosmo Bio Co. Ltd.), mouse monoclonal GAPDH antibody (1:10000, Biodesign), mouse monoclonal Hsp70 antibody (1:1000, Stressgen), rabbit polyclonal LC3B antibody (1:1000, Cell Signaling Technology) or rat monoclonal Hsp90 antibody (1:5000, Stressgen) overnight at 4°C. Membranes were washed three times for 10 min in TBST and then incubated with donkey anti-rabbit, anti-mouse or anti-rat IgG conjugated to horseradish peroxidase (1:5000; Jackson ImmunoResearch) for 1 hour. Membranes were washed three times each for 10 min, and protein expression was visualized by ECL treatment and exposure to film. The levels of total GFP-TDP_220-414_, phosphorylated-GFP-TDP_220-414_, Hsp70 and Hsp90 were normalized to their corresponding GAPDH bands to correct for protein loading.

### Statistics

Data from 3 separate experiments were analyzed by 1-way ANOVA, followed by Tukey's posthoc analysis.

## List of abbreviations

(ALS): amyotrophic lateral sclerosis; CMV: cytomegalovirus; FBS: fetal bovine serum; FTLD-U: frontotemporal lobar degeneration with ubiquitin-positive inclusions; GFP: green fluorescent protein; HMW: high-molecular weight; Hsp: heat shock proteins; PBS: phosphate-buffered saline; TDP-43: TAR DNA binding protein-43; UPS: ubiquitin-proteasome system

## Competing interests

The authors declare that they have no competing interests.

## Authors' contributions

YZ and YX performed experiments. YZ and TG performed data analysis and co-wrote the manuscript. TG contributed to the siRNA knock-down and solubility studies. LK and SY provided M17D cell line and assistance with the characterization of the cell culture model. LP conceived of the study, participated in its design and coordination and edited the manuscript. All authors read and approved the final manuscript.
